# Effect of quadriceps training at different levels of blood flow restriction on quadriceps strength and thickness in the mid-term postoperative period after anterior cruciate ligament reconstruction: a randomized controlled external pilot study

**DOI:** 10.1186/s12891-023-06483-x

**Published:** 2023-05-08

**Authors:** Xuefeng Li, Jinyu Li, Liang Qing, Haonan Wang, Huijun Ma, Peng Huang

**Affiliations:** 1 School of Sports Medicine and Rehabilitation, Beijing Sport University, Beijing, 100084 China; 2 College of Physical Education, Taiyuan University of Technology, Taiyuan, 030024 China

**Keywords:** Blood flow restriction training, Accelerated rehabilitation, Different level of restriction, Muscle strength, The thickness of the rectus femoris and vastus intermedius

## Abstract

**Background:**

More than 2 million anterior cruciate ligament (ACL) injuries occur worldwide each year. Most surgeons suggest that athletes and active persons with significant knee functional demands, including cutting motions, require and should be offered ligament reconstruction surgery. Despite concentrated rehabilitation efforts, deficits in quadriceps size and strength can persist for years after surgery. Blood flow restriction (BFR) training can help overcome disuse muscular atrophy in the mid-term postoperative period after anterior cruciate ligament reconstruction (ACLR) surgery. The purpose of this study was to evaluate the effects of quadriceps training with different levels of blood flow restriction on quadriceps strength and thickness of participants after ACLR.

**Methods:**

In this study, 30 post-ACL reconstruction participants were randomly divided into three groups (control, 40% Arterial Occlusion Pressure [AOP] and 80% AOP groups). All patients were subjected to different levels of BFR, combined with conventional quadriceps rehabilitation, for 8 weeks. Assessments included scaled maximal isokinetic knee extension strength at 60°/s and 180°/s, the sum of the thickness of the affected femoris rectus and vastus intermedius, Y-balance test performance, and International Knee Documentation Committee questionnaire responses before and after the intervention.

**Results:**

In total, 23 participants completed the entire study. The 80% AOP compression group showed an increase in quadriceps femoris muscle strength and muscle thickness (p < 0.01). As compared with the control group, outcome indicators in the 40% AOP and 80% AOP group were improved (p < 0.05). After 8 weeks of experimental BFR intervention, the results were better for the 80% AOP compression group than for the 40% AOP compression group in quadriceps peak torque to body weight at 60°/s and 180°/s angular velocity, as well as the sum of the thickness of the rectus femoris and vastus intermedius.

**Conclusion:**

The combination of BFR and low-intensity quadriceps femoris training can effectively improve the muscle strength and thickness of knee extensors in participants with ACLR and help reduce the difference between the healthy and surgical sides of the knee joint while improving knee-joint function. Choosing quadriceps training with 80% AOP compression intensity could provide the most benefits. Meanwhile, BFR can accelerate the rehabilitation process of patients and allow early entry into the next rehabilitation cycle.

**Registration:**

Trial registration Chinese Clinical Trial Registry, registration number ChiCTR2100050011, date of registration: 15/08/2021.

## Introduction

More than 2 million anterior cruciate ligament (ACL) injuries occur worldwide each year [1]. Most surgeons suggest that athletes and active persons with significant knee functional demands including cutting motions need and should be offered ligament reconstruction surgery [2]. Despite concentrated rehabilitation efforts, deficits in quadriceps size and strength can persist for years after surgery. However, ACLR surgery causes trauma to the knee joint, which then requires a period of rest and recovery after surgery [3]. In the process of postoperative rehabilitation, patients with ACLR face problems with quadriceps femoris muscle strength and function decline [4–9], leading to the delayed recovery of knee-joint function. The middle postoperative period for ACLR is 3–6 months after surgery. This stage is associated with the complete cessation of healing, the end of the proliferative period, and entry into the graft ligament phase. The restoration of muscle strength, balance, and basic coordination is the medium-term goal after surgery. This stage typically begins with simple bodyweight exercises, followed by a strength-based pattern that integrates resistance, balance, and coordination exercises. Nevertheless, despite continuous progress in transplantation and reconstruction techniques, the results of ACLR are still poor. Strength recovery in the quadriceps is essential in the rehabilitation of patients after cruciate ligament surgery, but early strength gains in this muscle are associated with improvements in knee function [10].

The current guidelines recommend that ACLR patients train at 60–100% intensity with a single maximum repetition (1RM) load to increase muscle strength, explosiveness, and endurance [11]. However, postoperative patients are limited by either not being able to meet the necessary loads or the need to protect the postoperative limbs. After anterior cruciate ligament reconstruction, muscle thickness can be significantly increased by combining blood flow restriction (BFR) with low-intensity resistance training [12, 13]. As reviewed by Scott et al. [14], blood flow restriction training (BFRT) can improve muscle strength and CSA after ACL reconstruction by affecting muscle fiber regeneration, stem cell proliferation, and metabolic stress. These effects are beneficial to healing and can potentially improve recovery and functional outcomes. Lambert et al. [15] found that BFR therapy can protect the entire body’s skeleton and muscles after surgery. In addition to providing greater muscle restoration than standard rehabilitation alone, the inclusion of BFR in ACL rehabilitation appears to have a protective effect on the bones. Also, ACLR postoperative grafts in the middle postoperative period are in the ligamentisation phase, during which the graft’s mechanical strength is rising phase. At this time, strength training should be performed to help the patient resume daily life and exercise.

We implemented this pilot study to evaluate the effects of quadriceps training with different levels of blood flow restriction on quadriceps strength and thickness of participants after ACLR. Additionally, the feasibility of the experiment will also be assessed. It was hypothesized that a combination of BFR and low-intensity quadriceps strength training can effectively improve the quadriceps atrophy, decreased muscle strength, and knee dysfunction that occurs in patients during mid-postoperative ACLR. Additionally, we investigated whether the level of BFR contributes to the variability in treatment outcomes.

## Methods

### Study design

**Fig. 1:**
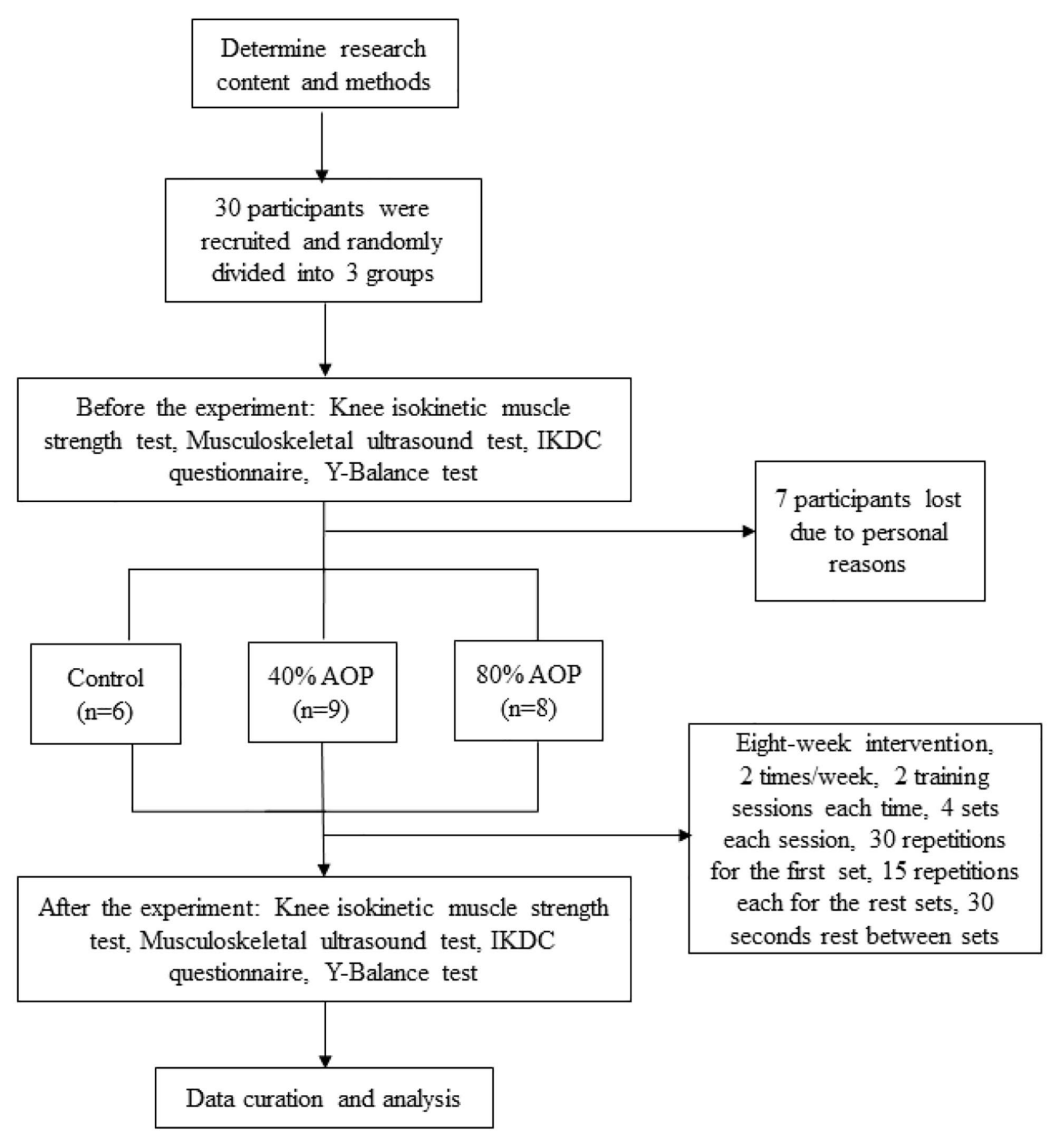
Experimental flow chart

This study was designed as a randomized controlled external trial. The trial was reviewed and approved by the Ethics Review Committee of Beijing Sport University, with approval number 2,021,068 H. The Chinese Clinical Trial Registry registration number is ChiCTR2100050011, and the date of registration is 15/08/2021. For further details regarding the exact experimental designs employed, please refer to the flow chart, as shown in Fig. 1.

### Setting

This trial was conducted between August 2021 and June 2022 in Haisikang Sports Medicine Clinic, Beijing, China.

### Participant recruitment and allocation

The four physical therapists recruited a community of individuals who underwent anterior cruciate ligament reconstruction (ACLR) at Haisikang Sports Medicine Clinic, Jianxingzhe Exercise Rehabilitation Center, and Peking University Third Hospital in Beijing via print and social media advertisements. The study’s directors adopted Excel software to randomly assign 30 participants into three groups comprising 10 participants each. The process of randomizing the grouping was performed using the RANDBETWEEN function in Microsoft Excel. A column containing a sequential numbering of the 30 participants was created, and a second column was added to generate a random number between 1 and 3 for each participant using the aforementioned RANDBETWEEN function. The output of this function was used to assign each participant to one of the three groups in a randomized fashion. The resulting groups were then sorted based on their assigned number and displayed accordingly, ensuring that each individual was placed into an unbiased and randomly selected group.

### Participants

A total of 30 participants from hospitals and rehabilitation institutions in Beijing who had undergone ACLR surgery were screened by completing the Physical Activity Readiness Questionnaire (PAR-Q) portion of the Physical Activity Questionnaire.

- Inclusion criteria: participants aged 18–40 years who underwent fork reconstructive surgery and were reconstructed using an autologous tendon graft, were beyond 8 weeks after reconstructive surgery, and had an active flexion motion of 100° on the operative side of the knee.
- Exclusion criteria: participants with a history of deep vein thrombosis, pregnancy, congenital heart disease, varicose veins, or contraindications to exercise.

During the 8-week experiment, seven participants withdrew from the study for physical, work, and other reasons, leaving 23 participants who completed the final experiment and were included in the statistical analysis. The basic information of the 23 participants is shown in Table 1.

**Table 1:** Group Characteristics (mean ± sd)

Group	n	Age (y)	Height (cm)	Weight (kg)	Time to Surgery (week)
Control	6	28.33 ± 5.19	172.33 ± 12.56	74.67 ± 23.09	20.17 ± 9.13
40% AOP	9	29.67 ± 3.97	171.33 ± 09.23	71.22 ± 11.72	22.78 ± 5.97
80% AOP	8	30.50 ± 5.26	175.87 ± 11.06	79.13 ± 12.78	22.25 ± 9.47

### Sample size

Sample size estimation was conducted using the G*Power software. To determine the appropriate sample size for our study, we consulted a previous meta-analysis that reported the effects of blood flow restriction training on muscle strength in clinical musculoskeletal rehabilitation [16]. Following this guidance, we conducted a power analysis assuming a mean difference and standard deviation between the groups, with α = 0.05 and β = 0.90, and equal sample sizes for the intervention and control groups. The power analysis and sample size calculation indicated that a minimum of 9 patients were required per group. To account for a projected dropout rate of 10%, we aimed to include 10 patients per group in the study.

### Procedure

Before inclusion in the experimental study, the participants were informed of the purpose and content of the study, and each participant provided written informed consent by signing a consent form. All participants were informed of their right to withdraw from the experimental intervention at any time during the course of the experiment. Physiotherapists were instructed to stop the exercise intervention immediately if any participant experienced significant discomfort during the intervention.

At baseline (PRE) and after 8 weeks of training (POST), we assessed scaled maximal isokinetic knee extension strength at 60°/s and 180°/s, the sum of the thickness of the affected the rectus femoris and vastus intermedius, Y-balance test performance, and International Knee Documentation Committee questionnaire responses. Self-reported adverse events were recorded throughout the protocol.

One-to-one intervention training was provided by four physiotherapists with over three years of experience. The physiotherapist underwent two consecutive two-hour training sessions as part of the study protocol. The first session took place one week before the start of the intervention, while the second session was held four weeks after the intervention had commenced. The training consisted of detailed explanations and demonstrations of the intervention program, including instructions on the proper use of the AirBands which was sourced from the VALD Performance company in Australia.

Pressurization method: We utilized AirBands as our compression equipment. After the participant has rested on the treatment bed for five minutes, the physical therapist operates a mobile phone app connected via Bluetooth to a compression device to automatically measure the participant’s maximum arterial occlusion pressure. The degree of compression for the exercise was measured and adjusted based on the measured arterial occlusion pressure of the participant. During the training process, after completing each exercise, the participant rested for 30 s, and the mobile phone software operated the equipment for deflation and pausing. After this 30-second rest, the pressurized belt was automatically inflated to the last set pressure value. This process was repeated until the training was complete.

Exercise protocols: During the eight-week intervention, all participants were required to complete a total of 16 training sessions, consisting of two sessions per week, with each session lasting approximately 60 min. Each training session included a 10-minute walking warm-up, followed by two quadriceps training exercises. Participants also completed stretching exercises for relaxation after each training session. During each training exercise, participants were instructed to complete four sets of 30 repetitions for the first set, and 15 repetitions for the remaining sets, with a 30-second rest between each set. The intensity of the exercises was adjusted based on individual participant needs and progression throughout the intervention. Male participants utilized green Thera-Band USA elastic bands, while female participants utilized red ones. The weight of the barbell bar employed for weight training was 20 kg. For males, the barbell bar was loaded with 10 kg on each end, whereas for females, the bar was utilized without additional weights. The load could be adjusted according to the participant’s requirements and the principle of adjustment was that 5 kg barbell weights could be added to each end of the bar. Table 2 below shows the intervention details.

**Table 2:** Template for intervention description and replication (TIDieR)

Checklist item	Details
Name of intervention for experimental/comparator	BFRT(Blood flow restriction training)
Rationale	BFRT can improve post-operative muscle atrophy and dysfunction due to the inability to train at high intensity.
Materials used in the intervention	AirBands which was sourced from the VALD Performance company in Australia.
Intervention procedures	Week 1 cycling exercise, straight-knee isometric resistance training with elastic band Week 2 straight-knee isometric resistance training with an elastic band, squat against the wall with a yoga ball Week 3 squat against the wall with yoga ball, self-weight lunge squat Week 4 self-weight lunge squat, up and down stairs Week 5 standing resistance knee extension with an elastic band, resistance squat Week 6 standing resistance knee extension, resistance squat Week 7 resistance squat, single-leg Bulgarian squat Week 8 resistance squat, resistance lunge walk
Provider	Four physiotherapists with over three years of experience
Mode of intervention delivery	One-to-one intervention training was provided by physiotherapists
Setting of intervention	Haisikang Sports Medicine Clinic
Dosage	All participants were required to complete a total of 16 training sessions, consisting of two sessions per week. Each training session included a 10-minute walking warm-up, followed by two quadriceps training exercises. Participants also completed stretching exercises for relaxation after each training session. During each training exercise, participants were instructed to complete four sets of 30 repetitions for the first set, and 15 repetitions for the remaining sets, with a 30-second rest between each set.
Tailoring	Male participants utilized green Thera-Band USA elastic bands, while female participants utilized red ones. The weight of the barbell bar employed for weight training was 20 kg. For males, the barbell bar was loaded with 10 kg on each end, whereas for females, the bar was utilized without additional weights. The load could be adjusted according to the participant’s requirements and the principle of adjustment was that 5 kg barbell weights could be added to each end of the bar.
Modifications	During the pilot, we adapted the original protocol to remove assessments on the Lysholm Knee Score and Single leg hopping test. The primary reasons for the change were some of the participants were unable to complete the jumping movement of the operated leg and the IKDC questionnaire was more sensitive and responsive to changes in knee function after ACL reconstruction. The primary outcome of this study, muscle strength, and muscle thickness was not affected.
Fidelity assessment	The statistical analysis included data only from participants who completed all 16 intervention training sessions

### Outcome measures

#### Quadriceps strength

Isokinetic concentric and eccentric knee extensor and flexor strength variables are reliable [17]. To perform the isokinetic test, the participant sat on a Biodex 3.0 dynamometer with a hip flexion of 90°. The distal end of the tibia, near the ankle, was fixed to the force arm of the dynamometer, and a Velcro belt immobilized the thigh and pelvis. The axis of rotation was set in the lateral femur condyle. To ensure maximum effort, the participant was familiarised with the experimental procedures and received visual feedback from the tester’s verbal motivation and the real-time stress display of the dynamometer. The participant performed three pre-experiments and began the test after a 5-minute break. During the test, the participant was flexed and extended to the maximum centripetal of each knee joint, which was repeated eight times at 60°/s and twelve times at 180°/s. Custom software was used to determine the maximum autonomous force of the affected limb in the test and, thus, the peak moment and total work.

#### Muscle thickness

Diagnostic musculoskeletal ultrasound can be used as an alternative tool for the rapid and effective measurement of quadriceps thickness (cross-sectional area) [18]. In this experiment, a color Doppler ultrasound diagnostic system (model Apogee 1000) was used for the thickness test. The participant assumed a supine position, with the hands naturally placed on both sides of the body. The lower limbs were completely placed on the bed, and the entire body was completely relaxed for 5 min before the measurement. With the thigh skin fully exposed and the lower limb muscles in a state of complete relaxation, the ultrasound probe was placed at the midpoint of the connection between the anterior superior iliac spines and the upper part of the patella, keeping the probe direction parallel to the connection and perpendicular to the surface of the rectus femur and using the couplant to fully contact the skin without compressing the soft tissues. The probe angle was fine-tuned until the ultrasound image was clearly visible and the muscles and fascia were clearly layered for easy marking and measurement. The depth of ultrasound detection was then adjusted to ensure that the rectus femoral muscle and its deep vastus intermedius muscle, including the femur, were within the range of the ultrasound images. The specific profile ranges of the rectus femoral muscle and the intermediate femoral muscle were determined for dot markings and connected with vertical measurement lines, according to the machine value, to derive the muscle thickness of the rectus femoral muscle and vastus intermedius muscle.

#### Knee joint function

At present, the 2000 International Knee Documentation Committee (IKDC) Scale is internationally recognized for its high reliability, effectiveness, and sensitivity in the assessment of ligament injuries, particularly ACL injuries and defects [19]. In this experiment, the participants were evaluated using a 2,000 IKDC subscale to determine the changes in the participant’s knee function before and after the experiment.

#### Knee joint stability

The Y-balance test (YBT) is a modified test for assessing upper and lower limb stability based on the star offset balance test. This test can reflect the participant’s lower extremity (or upper extremity) stability and left-right balance [20]. To perform the YBT test, the participant stood on the test platform with their hands on their hips and the toes of the injured leg aligned with the red starting line on the test platform. The other foot pushed the test board as far as possible forward, posterior medial, and posterior lateral, and then returned to the starting line. The maximum distance, which was accurate to 0.5 cm, the test board was pushed in various directions was measured, and the test was repeated three times. The leg support was changed, the test was repeated and the results were recorded.

### Statistical analysis

All data were expressed as means ± standard deviations (SDs). A linear mixed model with repeated measures was used to assess the data for the fixed factors of the trial (control group, 40% AOP group, and 80% AOP group) and time (primary and secondary outcomes). The level of significance was set at P < 0.05 for all data. The Bonferroni method was selected as the p-value correction method, and the corrected p-value was directly compared with 0.05. Statistical analyses were performed using SPSS 22.0 (SPSS Inc., Chicago, USA).

## Results

### Comparison of variability between the three groups

The results regarding the single-factor variance between groups were F = 8.108 and P < 0.01, suggesting a statistical difference in the peak torque at an angular velocity of 60°/s among the three groups. After the intervention, significant differences were detected between the control group (94.05 ± 17.62 N·m/kg) and the 40% AOP group (123.94 ± 28.13 N·m/kg) and 80% AOP group (152.04 ± 25.45 N·m/kg) (P < 0.05). Differences were also observed between the 40% AOP group (123.94 ± 28.13 N·m/kg) and the 80% AOP group (152.04 ± 25.45 N·m/kg) (P < 0.05).

The results regarding single-factor variance between the groups showed F = 14.674 and P < 0.01, suggesting the occurrence of a statistical difference in peak torque at an angular velocity of 180°/S between the three groups. After the intervention, significant differences were noted between the control group (73.28 ± 15.73 N·m/kg) and the 40% AOP group (102.06 ± 17.34 N·m/kg) and 80% AOP group (137.65 ± 25.54 N·m/kg) (P < 0.05). Significant differences were also detected between the 40% AOP group (102.06 ± 17.34 N·m/kg) and the 80% AOP group (137.65 ± 25.54 N·m/kg) (P < 0.05).

The results regarding single-factor variance between groups were F = 11.153 and P < 0.01, suggesting statistical differences in the sum of the thicknesses of the rectus femoris and intermediate femoris between the three groups. After the intervention, significant differences were observed between the control group (4.32 ± 0.70 cm) and the 40% AOP compression group (5.23 ± 0.84 cm) and 80% AOP compression group (6.26 ± 0.64 cm) (P < 0.05). Significant differences were also apparent between the 40% AOP group (5.23 ± 0.84 cm) and the 80% AOP group (6.26 ± 0.64 cm) (P < 0.05).

The results regarding single-factor variance between groups were F = 3.576 and P < 0.05, suggesting a statistical difference in IKDC scores between the three groups. After the intervention, the control group’s score (59.00 ± 10.79) was significantly different from the 80% AOP group’s score (75.38 ± 10.45) (P < 0.05). No significant differences were evident between the other groups (P > 0.05).

The Kruskal-Wallis test results were H = 8.036 and p (0.045) < 0.05, indicating a statistical difference in the two-sided difference YBT scores between the various compression groups, but the specific difference between these two groups requires further examination. The DunnTest command was used to perform the Dunn method and thus achieve a pairwise comparison of the three groups. The Bonferroni method was selected as the p-value correction method, and the corrected p-value was directly compared with 0.05. Significant differences were obtained between the control group and the 80% AOP compression group (P < 0.05), but no significant differences were detected for the other groups (P > 0.05). Detailed data are shown in Table 3.

**Table 3:** The specific differences in the two-sided difference scores for the YBT in the different compression groups

Variable	Case		
	Control	40% AOP group	80% AOP group
PT/BW (60°/s) (N·m/kg)	94.05 ± 17.62	123.94 ± 28.13	152.04 ± 25.45
PT/BW (180°/s) (N·m/kg)	73.28 ± 15.73	102.06 ± 17.34	137.65 ± 25.54
Thickness (cm)	4.32 ± 0.70	5.23 ± 0.84	6.26 ± 0.64
IKDC (%)	59.00 ± 10.79	65.11 ± 10.65	75.38 ± 10.45
YBT	0.15 ± 0.08	0.13 ± 0.10	0.05 ± 0.03
Variable	**P**		
	Control VS 40% group	Control VS 80% group	40% VS 80% group
PT/BW (60°/s) (N·m/kg) PT/BW (180°/s) (N·m/kg) Thickness (cm) IKDC (%) YBT	0.030* 0.013* 0.017* 0.295 0.534	0.000* 0.000** 0.000** 0.000** 0.021**	0.027* 0.001* 0.004** 0.000** 0.050

Data are expressed as mean ± SD and level of significance (P).

*P<0.05 for between-group variation; **P<0.01 for between-group variation.

AOP, Arterial Occlusion Pressure; PT/BW, Peak Torque to Body Weigh; Thickness, the thickness of the rectus femoris and vastus intermedius; IKDC, International Knee Documentation Committee; YBT, Y-balance test.

### Changes in participants’ indices before and after the experiment (paired)

The control group, that is, the 0% AOP pressure group, showed no improvement in the relative peak torque of the knee extensor muscle, the sum of the thickness of rectus femoris and vastus intermedius, or the IKDC score within the group before and after the experimental intervention.

**Fig. 2:**
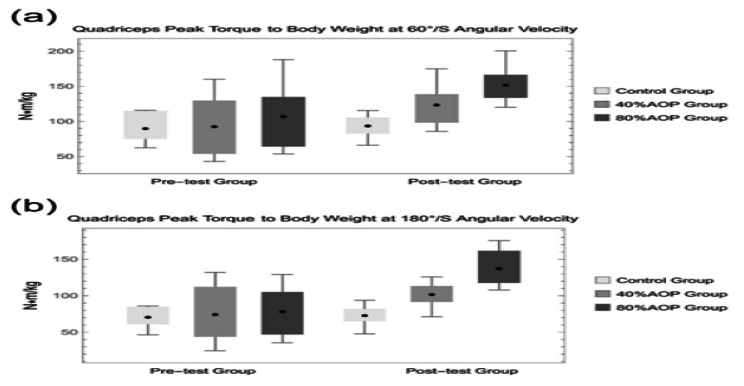
**(a)** Quadriceps peak torque to body weight at 60°/s angular velocity; **(b)** Quadriceps peak torque to body weight at 180°/s angular velocity

Figure 2(a) showed that the mean level of the relative peak torque of the knee extensor muscle group at 60°/s of the isometric apparatus increased from 93.02 ± 46.61 N·m/kg to 123.94 ± 28.13 N·m/kg in the 40% AOP group, as well as that the mean level of peak torque at an angular velocity of 60°/s was significantly higher after the intervention than before the intervention (P < 0.05). By comparison, in the 80% AOP group, the mean level of relative peak torque of the knee extensor muscle group at 60°/s of the isometric apparatus increased from 107.53 ± 46.75 N·m/kg to 152.04 ± 25.45 N·m/kg, and the mean level of peak torque at an angular velocity of 60°/s was significantly higher after the intervention than before the intervention (P < 0.01).

Figure 2(b) reveals an increase in the mean level of the relative peak torque for the knee extensor muscle group in the 40% AOP group at 180°/s of the isometric apparatus from 74.72 ± 39.93 N·m/kg to 102.06 ± 17.34 N·m/kg, as well as that the mean level of the peak torque at 180°/s angular velocity was significantly higher after the intervention than before the intervention (P < 0.05). In the 80% AOP group, the mean level of relative peak torque of the knee extensor muscle group at 180°/s of the isometric apparatus increased from 78.69 ± 34.47 N·m/kg to 137.65 ± 25.54 N·m/kg, as well as that the mean level of peak torque at an angular velocity of 60°/s was significantly higher after the intervention than before the intervention (P < 0.01).

**Fig. 3:**
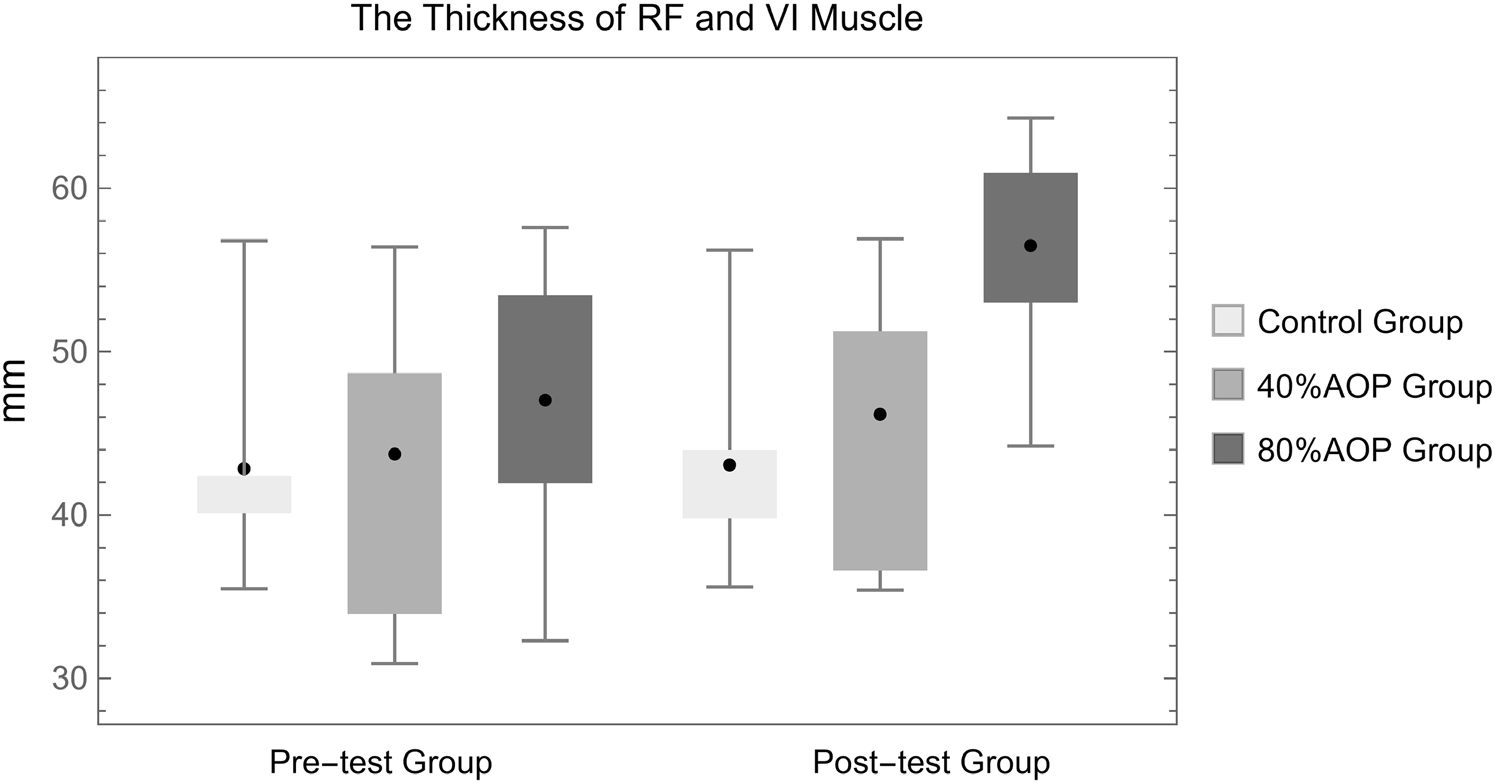
The thickness of the rectus femoris muscle and vastus intermedius

Figure 3 showed that, after eight weeks of experimental intervention, the posterior measurements of the experimental groups were significantly improved as compared to the premeasured. In the group with 40% AOP compression, the thickness of the rectus femoris and vastus intermedius on the operated side increased by 0.84 ± 0.13 cm, and the sum of the thickness of the rectus femoris and vastus intermedius increased from 4.38 ± 0.90 cm to 5.23 ± 0.84 cm. The mean sum of the thickness of the rectus femoris and vastus intermedius was significantly higher after treatment than before the intervention (P < 0.01). The mean sum of the thickness of the rectus femoris and vastus intermedius in the 80% AOP compression group increased from 4.71 ± 0.83 cm to 6.26 ± 0.64 cm, which was significantly higher after the experiment than before the intervention (P < 0.01).

**Fig. 4:**
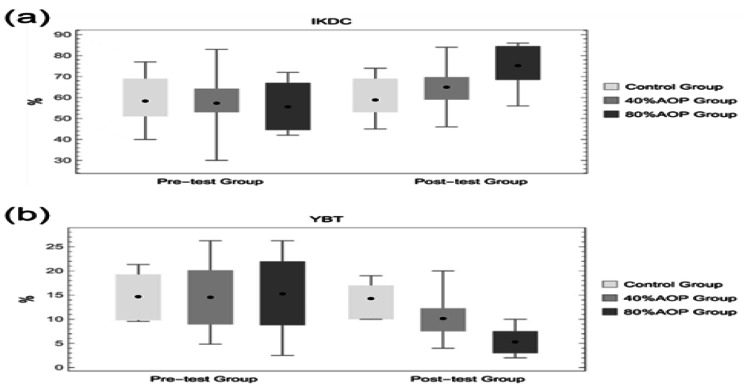
IKDC **(a)** and YBT **(b)** scale scores

The IKDC scale scores were used to assess the functional recovery of the participants’ knee joints. Figure 4(a) showed that participants in the 40% AOP compression group had a mean baseline IKDC score of 57.44 ± 14.03 before the intervention and a mean IKDC score of 65.11 ± 10.65 after the 8-week intervention. The difference in IKDC scores before and after the compression intervention was 7.67 ± 3.38 (95% CI for the difference: -11.93, -3.40). The mean post-intervention IKDC score was statistically higher than the mean pre-intervention score (P < 0.01). The scores of the participants in the 80% AOP pressurized group improved from 55.75 ± 12.13 to 75.38 ± 10.45 at the pre-intervention baseline, an improvement of 19.63 ± 7.05 points. The mean post-intervention IKDC score was statistically higher than the pre-intervention score (p < 0.01), as shown in Fig. 4(b).

The YBT test was performed on participants before and after 8 weeks of training, and the test scores were derived and calculated to compare the bilateral stability differences between the 30 participants in the pre-and post-experimental phases. Figure 4(b) showed that the mean level of bilateral differences in the participants in the 40% AOP pressurization group was 0.18 ± 0.09 before the intervention at baseline and 0.13 ± 0.10 after the 8-week intervention, representing a difference of 0.04 ± 0.02 (95% CI for the difference: 0.03, 0.06). The mean bilateral difference was statistically lower after the intervention than before the intervention (P < 0.01). The mean bilateral difference in participants in the 80% AOP pressurized group was 0.15 ± 0.08 at baseline before the intervention and 0.05 ± 0.03 after the 8-week intervention, representing a difference of 0.10 ± 0.06 (95% CI for the difference: 0.05, 0.15). The mean bilateral difference was statistically lower after the intervention than before the intervention (P < 0.01).

A more intuitive comparison of the specific change in various parameters before and after the experiment is provided by the data shown in Table 4.

**Table 4:** The detailed data before and after the experiment

Index	Group	Initial	Final	Improvement	The 95% difference in CI	t	p
Peak Torque to Body Weigh (60°/s)	Control	90.20 ± 21.53	94.05 ± 17.62	3.85	(-12.09, 4.39)	-1.201	0.283
	40%AOP	93.02 ± 46.61	123.94 ± 28.13	30.92	(-58.18, -3.67)	-2.616	0.031*
	80%AOP	107.53 ± 46.75	152.04 ± 25.45	44.51	(-45.57, -24.32)	-5.213	0.001**
Peak Torque to Body Weigh (180°/s)	control	70.98 ± 15.07	73.28 ± 15.73	2.3	(-7.71, 3.11)	-1.093	0.324
	40%AOP	74.72 ± 39.93	102.06 ± 17.34	27.33	(-51.97, -2.70)	-2.559	0.034*
	80%AOP	78.69 ± 34.47	137.65 ± 25.54	58.96	(-72.91, -45.01)	-9.993	0.00**
Thickness	Control	4.29 ± 0.72	4.32 ± 0.70	0.02	(-0.19, 0.14)	-0.368	0.728
	40%AOP	4.38 ± 0.90	5.23 ± 0.84	0.84	(-0.94, -0.75)	-19.847	0.00**
	80%AOP	4.71 ± 0.83	6.26 ± 0.64	1.55	(-1.96, -1.13)	-8.89	0.00**
IKDC	control	58.50 ± 13.32	59.00 ± 10.79	0.5	(-3.52, 2.52)	-0.425	0.688
	40%AOP	57.44 ± 14.03	65.11 ± 10.65	7.67	(-11.93, -3.40)	-4.148	0.003**
	80%AOP	55.75 ± 12.13	75.38 ± 10.45	19.63	(-25.52, -13.73)	-7.874	0.000**
YBT	Control	0.15 ± 0.05	0.15 ± 0.08	0	(-0.09, 0.08)	-0.104	0.921
	40%AOP	0.18 ± 0.09	0.13 ± 0.10	-0.04	(0.03, 0.06)	6.1	0.00**
	80%AOP	0.15 ± 0.08	0.05 ± 0.03	-0.1	(0.05, 0.15)	4.83	0.002**

Data are expressed as mean ± SD and level of significance (P).

The first two lines of data (60°/s and 180°/s) point to the Peak Torque to Body Weigh.

Thickness, the thickness of the rectus femoris and vastus intermedius.

IKDC, International Knee Documentation Committee.

YBT, Y-balance test.

## Discussion

This trial examined the effects of different levels of compression on differences in muscle strength, thickness, knee function, and stability during the middle period of ACL reconstruction. The main findings of this clinical study are as follows:

(1) Blood flow restriction, combined with low-intensity quadriceps training, can effectively improve the strength and girth of the knee extensor muscle group in ACLR patients.

(2) Blood flow restriction, combined with low-intensity quadriceps training, was effective in decreasing bilateral differences in the lower extremity on the YBT test. It also had a positive effect on improving knee function. This can accelerate the rehabilitation process of patients and help them enter the next rehabilitation cycle as soon as possible.

(3) The level of compression has a noticeable effect on muscle strength, circumference, bilateral knee joint differences, and knee function recovery in patients during mid-term ACLR. If we do not consider the discomfort during compression training, choosing quadriceps training with 80% AOP compression intensity can provide the most benefits.

### Muscle strength

The isokinetic muscle strength test [17] is the gold standard for reflecting muscle strength. In this experiment, the maximum spontaneous force of the knee joint at angular velocities of 60°/s and 180°/s before and after the intervention was measured by isokinetic machines. Participants in the control group experienced some improvement in maximum extensor muscle strength after 8 weeks of training intervention, but the difference was not statistically significant. The maximum strength of the knee extension at different angular velocities was significantly improved after the experiment. Simultaneously with the increase in the level of compression, the strength of the knee joints was increased by an escalating margin. The expected outcomes of the experiment were that 8 weeks of compression training would improve the patient’s quadriceps muscle strength and that the level of compression would affect the participant’s muscle strength improvement. The final results of the experiment met the original expectations. The results were also consistent with a randomized controlled trial conducted by Tennent et al. [21], and the stretching power of the BFR groups was greatly improved as compared with conventional therapy. The reason for the lack of a significant difference in the control group was that a large range of intensive training could not be performed due to the reabsorption phase of the transplanted ligament. Simple low-strength quadriceps muscle strength training performed twice per week is not sufficient to provide a good improvement in the number and size of quadriceps muscle fibers. The failure of the quadriceps to reach fatigue at the physiological level prevents higher levels of mechanical tension and increases cell metabolic pressure, failing to achieve significant improvement in knee extension strength in the control group.

Metabolic stress caused by vascular occlusion during pressure training in the experimental group and mechanical tension caused by resistance training or exercise led to synergistic increases in muscle hypertrophy and strength [22–24]. Under BFR conditions, type II white muscle fibers can be activated in lower load resistance training [25, 26], and the maximum strength of the quadriceps muscle can be improved. A higher level of blood flow restriction reduces venous reflux, while increasing the body’s metabolic pressure, resulting in the faster compression of the lower extremities and, thus, reaching fatigue levels more quickly; increased satellite cell proliferation, accompanied by the synthesis of muscle proteins, and an increase in muscle fiber size and muscle strength [27, 28]. Depending on the amount and intensity of exercise, the degree of neuromuscular activation and the degree of fatigue will vary with the relative stress of the BFR [29]. The degree of increase in the maximum strength observed in the present experiment tended to moderate with increasing pressure, suggesting that increased levels of vascular occlusion may weaken type II muscle fibers’ stimulation, muscle strength, and hypertrophy. By contrast, the participants’ strength, measured at an angular velocity of 180°/s, increased to a greater extent than the increase in arterial occlusion pressure during the experiment, suggesting that the fatigue-resistant work capacity of the knee extensor muscle group increased in the participants after the pressurization intervention. At the same time, the degree of pressurization increased the metabolic pressure of the body and the physiological adaptability of the knee extensor muscles to fatigue resistance after 8 weeks of gradually increased training. The BFR, combined with low-stress quadriceps training, improved the knee extension strength of patients in the mid-term period after ACL surgery.

### Muscle hypertrophy

Diagnostic musculoskeletal ultrasound can be used as a tool for the rapid and effective measurement of quadriceps circumference (cross-sectional area). In this experiment, musculoskeletal ultrasound was used to measure the sum of the thickness of the rectus femoral muscle and the middle femoris muscle before and after the intervention, as well as to determine the variation in the extensor knee muscle before and after the experimental intervention. After the intervention, the sum of the thickness of rectus femoris and vastus intermedius was improved to a certain extent in the control group, but the difference was not statistically significant. The experimental group showed significant improvements in the rectus femoral and intermediate femoral girths after 8 weeks of intervention. As the level of compression increased, the increase in thickness of rectus femoris and vastus intermedius continued to increase.

The expected outcomes of the experiment were that 8 weeks of compression training would increase the patient’s quadriceps thickness and that the level of compression would affect individual circumference values. The experimental results and research of Ryan et al. [24] have shown that BFR can stimulate muscle hypertrophy to the same extent as high-intensity resistance training. Abe’s study of nine men with restricted venous blood flow to the leg muscles demonstrated that muscle size was increased by 4–7% after walking training [30]. Moderate BFR can dramatically increase hypertrophic stimuli, such as metabolic stress, skeletal muscle swelling, and neuromuscular activation [31]. In a periodic resistance training program, blood flow restriction training increases muscle hypertrophy. The main causes of muscle enlargement are mechanical tension, metabolic stress, and muscle damage.

Schoenfeld [22] believed that mechanical tension was the most important mechanism of muscle enlargement and that enlargement occurs during the process of mechanical force conduction. When the muscle fibers contract, the inner muscle segments are shortened and protrude from the side. This physical stretching of the muscle cell membranes is usually sensed by stretching receptors and seen as a threat to cellular structures. This tension can lead to the activation of several myogenic channels, such as mTOR, MAPK, and calcium-dependent channels. The result of this tension is increased muscle synthesis. When the mechanical pressure is sufficient, satellite cells are activated and bind to myofibrils. The nucleus of the satellite cell can provide more protein assembly machines for synthesizing the proteins needed for muscle growth.

Metabolic stress is another well-known mechanism of muscle enlargement. It usually occurs when anaerobic glycolysis, a process that occurs when the body uses glycogen faster than oxygen consumption, causes lactic acid and other metabolites to accumulate inside the cell. In the case of resistance training, if the patient receives high-frequency, short-interval training, this mechanism is activated. This is also how BFR training works to promote muscle growth. The increased muscle circumference in the experimental group was associated with the accumulation of metabolites, such as lactate and hydrogen ions, as well as the osmotic effect that promotes the infiltration of water molecules into the cells, resulting in cell edema. This edema stretches the muscle cell membrane, leading to an increase in the thickness of the direct and intermediate femoral muscles.

Post-training metabolite accumulation can also cause muscle fatigue by reducing the release of calcium ions from the sarcoplasmic reticulum, as well as reducing the sensitivity of actin and myosin to calcium ions. Changes in calcium ions increase the activity of muscle motor units with higher activation thresholds; these are often associated with larger fibers that produce more tension, so the cell membrane is stretched, thereby increasing the muscle circumference. Compression training allows muscle fatigue, which reduces the rate of contraction, while providing sufficient time to promote bridging formation so that more tension is generated, thereby allowing more cells to be stretched. An increase in metabolic stress, which leads to an increase in mechanical stress, therefore promotes the growth of muscle girth. Experimental results and studies by Jeremy P. Loenneke et al. [32] have shown that, when the level of pressurization is below 210 mmHg, the metabolic pressure becomes greater as the degree of compression increases, leading to increased mechanical stress and a more pronounced effect on muscle hypertrophy. To maintain the cells in a steady state, the rate of lactic acid and hydrogen ion accumulation will continue to decrease, the metabolic stress will gradually decline and the effect of muscle hypertrophy will gradually flatten.

### Knee joint stability

The YBT is commonly used to assess knee function and the symmetry of knee control. In this experiment, the YBT was used to measure the farthest length of the anterior, posterior medial, and posterior lateral directions of the knee bilateral limb of the participant and to determine the change in the bilateral control difference between the knee joint before and after the experimental intervention. The bilateral stability of the knee joint was improved after the experiment. However, only the 80% AOP compression group showed a statistically significant difference before and after the intervention. The one-way ANOVA results indicated only significant differences between the control group and the 80% AOP compression group. The expected outcomes of the experiment were that 8 weeks of compression training would improve bilateral control differences in the knee joint and that the level of compression would affect the level of recovery. The results obtained were fundamentally in line with the original expectations. In a study of knee osteoarthritis by Ladlow et al. [33], 28 adults with lower extremity knee osteoarthritis who completed a 3-week intensive rehabilitation program (the LL-BFR group) showed a significant improvement in overall Y-balance test scores. Previous studies have reported adaptations in muscle strength and hypertrophy located near the application of pressure due to the pre-fatigue of the muscles under the cuff [34]. Enhanced stimulation of the hip muscle tissue (located proximal to the cuff) could explain the significant improvement in the Y-balance test composite score of the LL-BFR group as compared to the conventional RT group.

The YBT mainly examines the participant’s knee joint nerve control ability and the static and dynamic stability of the lower extremities. No significant increases were detected in quadriceps muscle strength or circumference in the control group after 8 weeks of intervention, so no significant increase was observed in knee static or dynamic stabilization capacity. Improved knee nerve control may reflect increased stability in adjacent joints. The experimental BFR training can affect the proximal and distal ends due to the pressure on the lower limbs [35], and the training activities will also exercise the gluteal muscles, hamstrings, and other muscles, thereby increasing the stability of the pelvic hip joint and other areas. Improving the stability of the hip joint can have a positive impact on the dynamic stability of the lower limbs.

The reason for the lack of a statistically significant difference between the 40% AOP compression group and the 80% AOP compression group may be a slower recovery from neuromuscular fatigue after training at higher BFR levels. The improvement in the degree of motion could help the participants to support their legs in squats in the YBT, and healthy legs can push the square farther anteriorly, posterior medially, and posterior laterally, thereby improving the test score of the affected side and reducing the difference value of the healthy side. Experimental intervention protocols, such as the single-leg Bulgarian squat, may also help improve walking/running mechanics, thereby improving the dynamic stability of the knee joint. The compression training of the quadriceps of the lower extremities can improve the static dynamic stability of the knee joint, and lower extremity quadriceps compression at 80% AOP can effectively reduce the difference between the healthy and unhealthy sides.

Because of this trial’s small sample size, there was a chance of Type I error which is the mistaken rejection of a null hypothesis as the result of a test procedure, and Type II error which is the mistaken failure to reject the null hypothesis as the result of a test procedure. For example, one individual making a large gain would lead to this. In this situation, the IKDC and YBT statistics may not be scientific enough as indicators (power < 0.8).

### Knee joint function

The IKDC scale is commonly used to assess functional recovery in the knee ligament. In this experiment, the IKDC knee joint questionnaire was used to investigate the recovery of knee joint function in the participants and determine the changes in knee joint function before and after the experimental intervention. Little improvement was observed in knee function recovery in the control group, whereas the knee joint function of the experimental group was significantly improved after the experimental intervention. Increasing the level of compression provided greater recovery of knee joint function. The expected outcomes of the experiment were that 8 weeks of compression training would improve the patient’s knee function and that the level of compression would affect the level of recovery. The results met the original expectations. The low-load training of the control group did not increase muscle strength, nor did it improve the stability of the knee joint. The reason for the improvement in knee function in the experimental group was the improvement in muscle circumference and the strength of the muscles around the knee after 8 weeks of intervention. Closed-chain exercises for the knee have a clinically reported association with enhanced knee function [36]. The static stability of the participant is also improved as strength increases, and this is reflected in a significant reduction in knee weakness after the intervention.

Interventions in quadriceps training exercises, including wall yoga ball squats [37] and Bulgarian split squats [38], can enhance knee dynamic balance. As the flexion of the knee improves mobility, the degree of knee stiffness is also reduced. Studies have shown that BFR exercises for patients with knee pain can significantly reduce such knee pain. Improvements in quadriceps strength and thickness increased the participants’ likelihood of participating in the exercise. All the positive improvements mentioned above have had a positive impact on improving knee function.

### Limitations

During the pilot, we adapted the original protocol to remove assessments on the Lysholm Knee Score and Single leg hopping test. It was believed that this deviation from the protocol was justified. The primary reasons for the change were some of the participants were unable to complete the jumping movement of the operated leg and the IKDC questionnaire was more sensitive and responsive to changes in knee function after ACL reconstruction [19, 39]. This may therefore cause some deviation from the experimental assessments. However, the main indicators of the test, muscle strength, and muscle thickness, were not affected.

We did not blind participants or providers. This is because the limbs were relatively sensitive to changes in pressure levels. In addition, the providers needed to set the appropriate parameters for the participants according to their grouping and through the device. Furthermore, it is imperative to acknowledge the potential impact of the lack of blinding of outcome assessors on the study’s results. One limitation could be that the assessment of the outcomes may not be standardized across all participants due to the variability in the perception of the outcome assessor.

Because of this study’s small sample size, there was a chance of Type I error which is the mistaken rejection of a null hypothesis as the result of a test procedure, and Type II error which is the mistaken failure to reject the null hypothesis as the result of a test procedure. For example, one individual making a large gain would lead to this. In this situation, the IKDC and YBT statistics may not be scientific enough as indicators (power < 0.8).

## Research conclusions

(1) Blood flow restriction, combined with low-intensity quadriceps training, can effectively improve the knee extensor muscle strength and girth of patients with ACLR.

(2) Blood flow restriction, combined with low-intensity quadriceps training, can help reduce the difference between the affected and normal sides of the knee joint of ACLR patients and improve knee function, which can accelerate the patients’ rehabilitation process and help them enter the next rehabilitation cycle as soon as possible.

(3) The level of compression has a clear effect on the muscle strength, circumference, bilateral knee joint differences, and knee function recovery of patients during mid-term ACLR. If we do not consider the discomfort involved in the process of pressure training, the maximum benefit can be achieved by choosing an 80% AOP compression intensity for quadriceps femoris training.

## Research advice

(1) Patients in medium-term post-ACLR are recommended to choose a 40–80% AOP compression training level for daily rehabilitation training to accelerate recovery and return to life and exercise as soon as possible.

(2) Blood flow restriction at a certain level was observed to have some positive effects on the balance of the bilateral knee joints. Future studies could attempt to combine compression training with proprioceptive training or neuromuscular control training to determine whether different levels of compression training can promote the recovery of proprioception or the balance of the bilateral knee joints in the postoperative population.

## Data Availability

The data used and analyzed for the current study are available from the corresponding author upon reasonable request.
